# Identification of quantitative trait loci (QTL) controlling resistance to pea weevil (*Bruchus pisorum*) in a high-density integrated DArTseq SNP-based genetic map of pea

**DOI:** 10.1038/s41598-019-56987-7

**Published:** 2020-01-08

**Authors:** Thais Aznar-Fernández, Eleonora Barilli, María J. Cobos, Andrzej Kilian, Jason Carling, Diego Rubiales

**Affiliations:** 1 Institute for Sustainable Agriculture, CSIC, Córdoba, E-14004 Spain; 2 Diversity Arrays Technology Pty Ltd, University of Canberra, Kirinari St. Bruce, ACT2617 Australia

**Keywords:** Quantitative trait, Biotic

## Abstract

Pea weevil (*Bruchus pisorum*) is a damaging insect pest affecting pea (*Pisum sativum*) production worldwide. No resistant cultivars are available, although some levels of incomplete resistance have been identified in *Pisum* germplasm. To decipher the genetic control underlying the resistance previously identify in *P. sativum* ssp. *syriacum*, a recombinant inbred line (RIL F_8:9_) population was developed. The RIL was genotyped through Diversity Arrays Technology PL’s DArTseq platform and screened under field conditions for weevil seed infestation and larval development along 5 environments. A newly integrated genetic linkage map was generated with a subset of 6,540 markers, assembled into seven linkage groups, equivalent to the number of haploid pea chromosomes. An accumulated distance of 2,503 cM was covered with an average density of 2.61 markers cM^−1^. The linkage map allowed the identification of three QTLs associated to reduced seed infestation along LGs I, II and IV. In addition, a QTL for reduced larval development was also identified in LGIV. Expression of these QTLs varied with the environment, being particularly interesting QTL *BpSI.III* that was detected in most of the environments studied. This high-saturated pea genetic map has also allowed the identification of seven potential candidate genes co-located with QTLs for marker-assisted selection, providing an opportunity for breeders to generate effective and sustainable strategies for weevil control.

## Introduction

Pea (*Pisum sativum* L.) is the second most cultivated temperate grain legume in the world and the first in Europe^1^. Its use extends to food and feed^2^. As most crops, pea can be damaged by a range of pests and diseases^3^. One of the most intractable and harmful pests worldwide is pea weevil (*Bruchus pisorum* L., Coleoptera: Bruchidae, *Bp*) causing seed yield losses of up to 50%^4,5^. *Bp* is a strict monophagous insect^6^ whose adults remain dormant in winter and reactivate when temperatures increase in coincidence with pea blooming. Females lay eggs on young pea pods and emerging larvae penetrate through the pods into the seeds, eating the cotyledon and finally moulting inside the seeds^7^. *Bp* control by insecticides is complicated because most of the insect life cycle is completed inside the seeds. Consequently, for the effectiveness of chemical treatments a constant monitoring on field is required to adjust the spray timing with *Bp* oviposition^8^. In addition, postharvest fumigations in storehouses might be needed to reduce the emergence of *Bp* that are hibernating or developing inside the seeds^9^. Because of the high ecologic and economic cost of chemical control, other strategies such as biological control with parasitoids^10,11^, early sowing, removal of crop residues^12^ or intercropping^13^ have been attempted. Unfortunately none of them provided sufficient level of control so far. This emphasize the need to develop *Bp* resistant cultivars for a more economic, ecologic and efficient *Bp* management. Resistance has been identified in germplasm accessions of *P. sativum* and wild relatives^12^. Out of these, resistance of *P. sativum* ssp. *syriacum* accession P665 showed highest and more stable levels of *Bp* resistance in field screenings^14^. This resistance was confirmed under controlled conditions, showing to be a combination of antixenosis and antibiosis mechanisms resulting in reduced seed infestation and retarded larval development^15^. This accession was previously identified as resistant to a number of other stresses such as ascochyta blight (*Didymella pinodes*)^16^, broomrape (*Orobanche crenata*)^17^, pea aphid (*Acyrtosiphon pisum*)^15^, and drought^18^, and therefore it was early introduced in the crossing program and extensively used in the breeding program at the Institute for Sustainable Agriculture, CSIC (Córdoba, Spain). In addition, a recombinant inbred line (RIL) population was generated from the cross with pea cv. Messire that was used in previous genetic studies^19^. The RIL is nowadays in an advanced generation (F_8:9_), avoiding the possible resulting distortion and is considered a valuable resource to unravel genetics of *Bp* resistance upon new high throughput marker deep genotyping.

The use of molecular markers linked to resistance genes seems to be an affordable and competent way to reduce efforts, time of evaluations, economic costs and do more efficient and effective the traditional plant breeding programs developed for pest control^3^. In order to achieve reliable information regarding the genomic regions involved in resistance, the precision in trait scoring and the availability of high density genetic maps are crucial. In this scenario, the Diversity Arrays Technology (DArT) in combination with next-generation sequencing platforms^20,21^ known as DArTseq^TM^, provides a good choice as a high throughput marker genotyping platform that can develop a relatively large number of polymorphic markers to build dense genetic maps with low-cost investments^22^.

Therefore, the objectives of the present study were the development of the first integrated high-density DArTseq based genetic linkage map of the interspecific *P. sativum* ssp. *syriacum* (P665) × *P. sativum* ssp. *sativum* (cv. Messire) RIL F_8:9_ population, as well as the identification of regions in the pea genome controlling *Bp* seed infestation and larval development.

## Material and Methods

### Plant materials

The population used in the study was composed of 108 recombinant inbred lines (RILs) families (F_8:9_) derived by a single seed method from a cross between the *Bp* resistant *Pisum sativum* ssp*. syriacum* accession P665 and the susceptible *P. sativum* ssp. *sativum* cv. Messire.

Response to *Bp* infestation of the RIL population together with their parental lines was studied under field conditions in a total of five environments (combination of locations and years), as follows: Córdoba (CORD) during growing seasons 2014–2015 and 2015–2016, Escacena del Campo (ESC) during growing seasons 2013–2014 and 2014–2015 and Espiel (ESP) in growing season 2015–2016 (Table 1). Field assays were designed following an incomplete block design with four repetitions. Experimental unit consisted in a 50 cm long row where 7–10 seeds for a line were sown. Rows were separated 50 cm between each other. In order to ensure a homogenous germination, seeds were previously scarified. No pesticide or herbicide was applied on trials and only mechanical weeding was done. In order to ensure a high and uniform infestation, 100 *Bp* adults (obtained from infested seeds of previous seasons) were freed in each field by middle March, when natural infestation starts^14^.

**Table 1:** Environmental conditions of the five trials tested during the study (sites and years).

Environment	Location	Season	Av. Temp (°C)	Av. Humidity (%)	Accu. Rainfall (mm)	Accu. Rad (W/m^2^)
ESC1	Escacena del Campo	2013–2014	15.4	72.27	376.4	16.6
ESC2	Escacena del Campo	2014–2015	15.2	66.1	130.0	17.7
CORD1	Córdoba	2014–2015	15.0	66.8	164.1	17.4
CORD2	Córdoba	2015–2016	15.0	69.9	354.2	16.1
ESP	Espiel	2015–2016	12.6	71.5	251.6	15.6

The parameters (Av. T: average temperature; Av humidity: average humidity; Accu. rainfall: accumulated rainfall; Accu.Rad: accumulated radiation) are given for the crop season (from sowing till harvest date).

**Figure 1:**
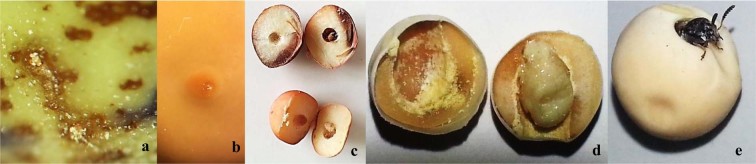
Seed infestation and larval stages (LS) of *Bruchus pisorum* (*Bp*) in parental lines, (**a**,**b**) holes of *Bp* larvae penetration (**a**) resistant parental line P665; (**b**) susceptible parental line cv. Messire; (**c**,**d**) opening of the seeds thorough the cotyledon to check the presence or absence of *Bp* larva inside seeds and LS; (c) LS2; (**d**) LS4; (**e**) *Bp* adult emergence from Messire, LS5.

Plants were manually harvested at maturity, threshed and seeds stored at 4 °C. Seed infestation (SI) was assessed in all environments, whereas larval development (LD) was assessed on CORD and ESP only. SI and LD were assessed by opening through the cotyledons of 100 seeds randomly taken from each replication^14^. SI was calculated as the percentage of seeds showing infestation at any stage of development. For LD calculation, larval stage (LS) need to be previously assessed in each seed following the visual scale proposed by Clement *et al*.^4^ with slight modifications, where: LS1 = first instar-larval penetration, 0–5% cotyledon eaten; LS2 = 6–25% cotyledon eaten; LS3 = 26–60% cotyledon eaten, second to fourth instar; LS4 = extensive damage, pre-pupa; LS5 = adult (Fig. 1). Subsequently, a larval development (LD) index was calculated as follows:

$$\,{\rm{LD}}=\frac{{\sum }^{}{\rm{LS}}\times {\rm{Ni}}}{5\times {\rm{Nt}}}\times 100$$

where LS is the larval stage, Ni is the number of seeds at each LS and Nt is the amount of Ni for each treatment, 5 showed the number of the total stages from the visual scale used in this study.

Analyses for phenotypic traits were made by using Statistix 10 ® (Analytical Software, Tallahassee, USA). The percentage data for SI was Ln transformed before subjecting to analysis of variance (ANOVA) in order to follow a normal distribution. Data for LD was analysed using generalized linear model (GLM) with one way analysis of variance (ANOVA). Pearson’s linear correlation was calculated to study the possible relationship between the parameters evaluated.

### Genetic map development

#### DNA extraction and quantification

Seedlings of all lines were grown under controlled conditions at CSIC Córdoba. Around 1 g of young leaf tissue from the 3^rd^-4^th^ node of each plant was excised, immediately frozen in liquid nitrogen and stored at −80 °C. Genomic DNA was isolated from fresh and young leaves of plants using a modified cetyltrimethylammonium bromide (CTAB)/chloroform/isoamylalcohol method^23^. DNA quantification was done by agarose gel electrophoresis (0.8%), and it was adjusted to 50 ng µl^−1^ for DArT and SNP genotyping.

#### Genotyping by DArTseq technology

A high-throughput genotyping method using the DArTseq™ technology at Diversity Arrays Technology Pty Ltd (Canberra, Australia) was employed to genotype the RIL population. Essentially, DArTseq™ technology relies on a complexity reduction method to enrich genomic representations with single copy sequences and subsequently perform next-generation sequencing using HiSeq. 2000 (Illumina, USA). DArTseq detects both SNPs and presence–absence sequence variants, collectively referred to as DArTseq markers^21^. DNA samples are processed in digestion/ligation reactions^20^, but replacing a single *PstI*-compatible adapter with two different adapters corresponding to two different restriction enzymes (RE) overhangs. The *PstI*-compatible adapter was designed to include Illumina flowcell attachment sequence, sequencing primer sequence and staggered, varying length barcode region. The reverse adapter contained the flowcell attachment region and *Mse*I-compatible overhang sequence. Only “mixed fragments” (*PstI*–*MseI*) were effectively amplified in 30 rounds of PCR using the following reaction conditions: 1 min at 94 °C for initial denaturation, 30 cycles each consisting of 20 s at 94 °C for denaturation, 30 s at 58 °C for annealing, 45 s at 72 °C for extension and finally a 7 min extension step at 72 °C. After PCR, equimolar amounts of amplification products from each sample of the 96-well microtiter plate were bulked and applied to c-Bot (Illumina) bridge PCR followed by sequencing on Illumina Hiseq. 2000. The sequencing (single read) was run for 77 cycles. Sequences generated from each lane were processed using proprietary DArT analytical pipelines. In the primary pipeline, the FASTQ files were first processed to filter poor-quality sequences, applying more stringent selection criteria to the barcode region compared to the rest of the sequence. Thus, the assignments of the sequences to specific samples carried in the “barcode split” step are more consistent. Approximately 2,500,000 (around 7%) sequences per barcode/sample are used in marker calling. Finally, identical sequences are collapsed into “fastqcall files”. These files were used in the secondary pipeline for DArT P/L’s proprietary SNP and SilicoDArT (Presence/Absence Markers in genomic representations) (present = 1 vs. absent = 0) calling algorithms (DArTsoft14). The analytical pipeline processed the sequence data.

The parameters used for quality control at the time of selecting high-quality SilicoDArT and derived SNPs markers^20^ for genetic mapping were: the reproducibility of 100%; the overall call rate (percentage of valid scores in all possible scores for a marker) over 95%; the polymorphic information content (PIC) between 0.3 and 0.5 and the Q value (the logarithm of the minimum false discovery rate at which the test may be called significant) above 2.5.

#### Linkage map and QTL mapping

The scores of all polymorphic DArTseq and SNP markers were converted into genotype codes (“A”, “B”) according to the scores of the parents. Linkage groups (LG) were obtained using the software JoinMap version 4.1^24^. The maximum likelihood mapping algorithm, which was optimised for constructing dense genetic maps using this software^25^, was first used for grouping all of the polymorphic markers. Then, the method of regression mapping^26^ was used for map construction with approximately 1,000 markers with appropriate genetic distance and the marker position, and the order of markers for three rounds to merge the tightly adjacent markers into bins. The markers in adjacent loci with genetic distance below 0.2 cM were classified into a bin during the first two rounds of mapping. Moreover, one marker with sequence information and with the least missing genotype from each bin was chosen as a “bin representative” for the next round of genetic mapping. In a third round of mapping, the makers in adjacent loci pairs with genetic distances below 0.1 cM were classified into a bin to avoid incorrect classification when the markers were decreased in the map. The Kosambi mapping function^27^ was used to convert recombination frequencies into map distances, and only “Map 1” was used for further analysis. The linkage groups maps of each chromosome were drawn and aligned using MapChart v2.3^28^. Segregation distortion was determined through a χ^2^ test for goodness-of-fit to the expected 1:1 ratio. Markers significantly deviating from Mendelian segregation were excluded in the first mapping round, according with Barilli *et al*.^22^. A minimum log-of-odds (LOD) score threshold of 3 and a maximum recombination fraction of 0.4 were applied as general linkage criteria to establish linkage groups (LGs). DArT markers were named with the numbers corresponding to unique clone ID following Kilian *et al*.^20^.

SNP markers previously mapped in this RIL were used as “anchor” markers to find the correspondence between this new pea map with previously published versions^19,29^ and to assign the *P. sativum* ssp. *syriacum* × *P. sativum* linkage groups to previously described *P. sativum* chromosomes. For the same purpose, the sequences from DArTseq-derived markers were compared with *Cicer arietinum* and *Medicago truncatula* genomic backbones by using Phytozome v.12 (https://phytozome.jgi.doe.gov/pz/portal.html) to perform a synteny analysis using three parameters recently defined by Salse *et al*.^30^ These parameters increase the stringency and significance of BLAST sequence alignment by parsing BLASTX results and rebuilding HSPs (High Scoring Pairs) or pairwise sequence alignments to identify accurate paralogous and orthologous relationships. This analysis allowed searching for sequence similarity-based homology between legume species providing an alternative approach to finding correspondence between linkage groups.

QTL analysis was conducted using composite interval mapping (CIM) and multiple interval mapping (MIM) in Windows QTL Cartographer v2.5^24^. Markers to be used as cofactors for CIM were selected by forward–backward stepwise regression. The number of markers controlling the genetic background in CIM was set to five. A threshold for the detection of a QTL was fixed at a LOD value of 3. For each LOD peak, the 1-LOD support intervals were calculated^29^. To obtain more precise information on QTL effects and positions, MIM was used by considering the CIM results obtained for the trait as initial QTL. Main additive effects were tested for significance using the Bayesian information criterion (BIC)^29^. The final main additive QTL effects and the R2 values of the model were then estimated. Skewness and Kurtosis coefficients were calculated following Lynch and Walsh procedure^31^.

The databases Unigene (http://bios.dijon.inra.fr/FATAL/cgi/pscam.cgi)^32^ and Uniprot (http://www.uniprot.org) were used for the identification of potential candidate genes linked to the genomic regions involved in weevil resistance.

### Ethical standards

The authors state that all experiments in the study comply with the ethical standards in EU.

## Results

### SI and LD assessment

**Figure 2:**
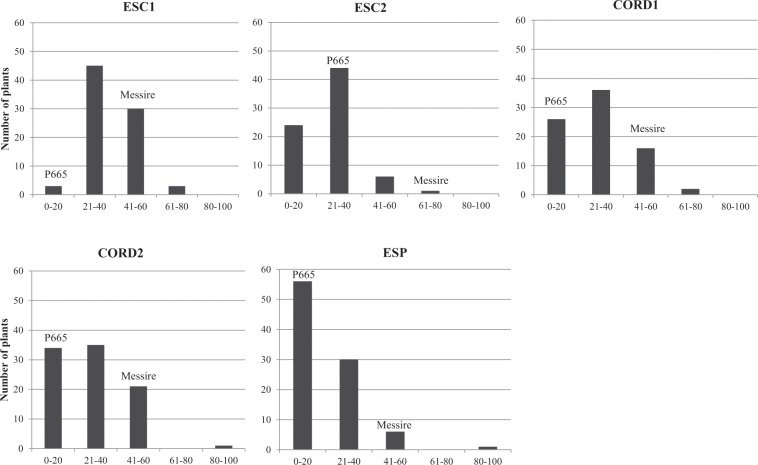
Frecuency distribution among RIL population lines (F_8:9_) derived from P665 × Messire cross in response to *Bruchus pisorum* seed infestation (%SI) under field conditions in five environements (ESC1: Escacena del Campo 2013–2014; ESC2: Escacena del Campo 2014–2015; CORD1: Córdoba 2014–2015; CORD2: Córdoba 2015–2016; ESP: Espiel 2015–2016). Position of resistant and susceptible parental lines (P665 and Messire, respectively) are shown for each environment.

Percentage of seed infestation (SI) assessed under field conditions showed different responses between parental lines in all environments, confirming previous findings^14^. Analysis of variance of each trial revealed significant genotypic effects for SI within the RIL population (P < 0.05) (Fig. 2). The parental accessions showed contrasting SI responses, with P665 being significantly more resistant (SI < 22%) than Messire (SI ranging between 50–75%) in all environment tested (P < 0.05) (Fig. 2). Distribution of residuals after analysis of variance was normal in each environment according to Lilliefors normality test (P > 0.05). Variances of genotypes and replicates were homogeneous according to Bartlett’s test (P > 0.05). The coefficient of Skewness under field conditions was of 0.77, 0.85, 1.04, 1.48 and 1.79 in environments ESC1, CORD1, ESC2, CORD2 and ESP, respectively, showing skewness towards reduced SI. SI values ranked between 0–100% and 4–85% depending on the year or the location under study (Table 2). They did not differ from the normal distribution, confirming the quantitative inheritance of the partial resistance. In addition, transgressive segregants with increased resistance and susceptibility compared with the parentals were observed for SI resistance criteria over years and conditions. Average values for SI measured on the whole RIL population showed differences between locations and years, being higher in ESC1 (38.2%) and lower in ESP (22.9%) (Table 2).

**Table 2:** Response to *Bp* infestation: mean values, minimum and maximum values of seed infection (SI) and larval development (LD) measured on the RIL population and parental lines at the different environments.

Environment	SI (%) Mean ± SE	LD Mean ± SE	SI (%) Min/Max	LD Min/Max
ESC1				
RIL	38.20 ± 1.3	nd	4.0/84.6	nd
P665	4.66 ± 0.3		4.0/5.0	
Messire	54.84 ± 0.6		50.0/58.8	
ESC2				
RIL	27.10 ± 1.3	nd	0.0/81.8	nd
P665	21.5 ± 1.3		20.0/25.0	
Messire	70.0 ± 1.78		67.0/75.0	
CORD1				
RIL	28.16 ± 1.7	70.9 ± 2.2	0.0/84.6	
P665	5.31 ± 2.7	16.5 ± 6.1	0.0/10.6	12.0/22.2
Messire	53.51 ± 6.7	70.0 ± 0.3	50.0/61.5	68.0/72.0
CORD2				
RIL	31.33 ± 2.5	55.4 ± 1.4	0.0/100.0	
P665	8.3 ± 2.4	15.33 ± 0.3	0.0/15.0	14.0/19.0
Messire	59.1 ± 1.1	71.26 ± 1.9	57.0/61.1	67.5/73.8
ESP				
RIL	22.92 ± 2.2	62.8 ± 1.7	0.0/100.0	
P665	3.93 ± 2.4	19.26 ± 1.8	0.0/10.0	18.0/22.6
Messire	56.5 ± 2.4	77.84 ± 3.7	50.0/61.0	68.7/86.2

**Figure 3:**
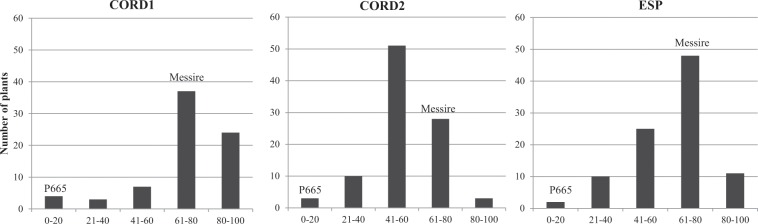
Frecuency distribution among RIL population lines (F_8:9_) derived from P665 × Messire cross in response to *Bruchus pisorum* larval development (LD) under field conditions in three environements (CORD1: Córdoba 2014–2015; CORD2: Córdoba 2015–2016; ESP: Espiel 2015–2016). Position of resistant and susceptible parental lines (P665 and Messire, respectively) are shown for each environment.

Parental lines showed also contrasting larval development (LD) values in all environments evaluated, being always <20 for P665 and >70 for cv. Messire (Fig. 3). LD values ranked between 5–89% and 12–92% depending on the year or the location under study (Table 2). LD did not follow a normal distribution (Lilliefors normality test; P < 0.05). The coefficient of Skewness for LD was of −1.08, −0.89 and −1.77 in CORD1, CORD2 and ESP, respectively, showing skewness towards high LD, with several transgressive RIL families showing higher LD values than the susceptible parental line, Messire (Fig. 3).

Pearson’s linear correlation coefficients between SI values evaluated in the field within years and locations were generally positively correlated and highly significant (Table 3). The same trend was also observed for LD values.

**Table 3:** Pearson’s linear correlation coefficients along the five environments between seed infestation (SI%) and larval development (LD).

	SI ESC1	SI ESC2	SI CORD1	SI CORD2	SI ESP	LD CORD1	LD CORD2
SI ESC2	0.52***						
SI CORD1	0.65***	0.43***					
SI CORD2	0.56***	0.61***	0.60***				
SI ESP	0.44***	0.55***	0.50***	0.40***			
LD CORD1	—	—	0.60***	0.30**	0.32**		
LD CORD2	—	—	0.44***	0.61***	0.42***	0.38***	
LD ESP	—	—	0.66***	0.41***	0.48***	0.63***	0.62***

***Significant at P = 0.001; **significant at P = 0.01; *significant at P = 0.05; ns = not significant.

### Construction of linkage maps

**Figure 4:**
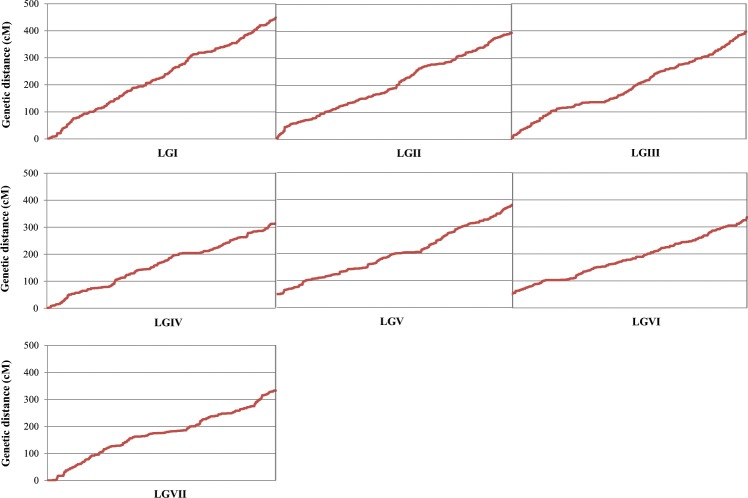
Distribution of DArT-seq-based and no-DArT-seq markers within each linkage group (LG) forming the interspecific *P. sativum* ssp. *syriacum* (P665) × *P. sativum* ssp. *sativum* (cv. Messire) cross derived map. The *x* axis shows the linkage group (LG) and the *y* axis shows the genetic distance (cM).

A total of 12,012 high-quality SilicoDArT and 14,880 SNPs markers were identified, for a total of 26,892 DArT-derived markers. Of these, a set of 6,447 markers (24.3%) were selected for mapping after a severe quality filtering. The mapping dataset was complemented with 93 SNP markers previously mapped in this RIL population^29^. All non-DArT derived markers mapped in the expected LGs according with previous publications ^19,29,33^. Markers were distributed across 7 LGs using LOD thresholds ranging from 3 to 10 and a recombination frequency (r) threshold <0.4 (JoinMap vs. 4). Each assigned group included at least five markers common to other published *P. sativum* genetic maps (Table 4, Fig. 4).

**Table 4:** Map features of P665 × Messire linkage map, including individual linkage group characteristics and their correspondence to *P. sativum* linkage groups and chromosomes.

Linkage group	DArT-seq markers	Other markers	Unique position	Distance (cM)	Average gap distance (cM)	Larger gap (cM)	Trimmed sequences		Corresponding pea chromosome
							*Medicago truncatula*	*Cicer arietinum*	
LGVII	705	6	187	332.88	1.78	6.38	24	22	1
LGIV	898	15	223	313.71	1.41	7.40	24	27	2
LGI	1,280	26	326	448.42	1.37	6.32	28	33	3
LGIII	936	11	263	396.88	1.51	7.37	26	19	4
LGVI	886	5	188	286.38	1.52	7.86	11	24	5
LGV	759	20	240	330.60	1.38	6.31	17	17	6
LGII	983	11	272	394.78	1.45	7.37	28	38	7
*Total*	*6,447*	93	*1,699*	*2,503.65*	*1.49*	*7.01*	158	180

The newly constructed integrated genetic linkage map of *P. sativum* ssp. *syriacum* × *P. sativum* ssp. *sativum* covered a total length of 2,503.6 cM, with an average density of 2.61 markers cM^−1^ and an average adjacent-marker gap distance of 1.49 cM (Table 4). The total number of mapped loci per LG ranged from 705 on LGVII to 1,280 on the LGI, and the average was of 921 loci LG^−1^. The longest individual LG map was for the LGI (448.4 cM), the shortest was for the LGVI (286.4 cM) (Table 4), and the average LG length was 357.7 cM. The density of markers in the individual LGs ranged from 2.12 markers cM^−1^ in the LGVII to 3.09 markers cM^−1^ in the LGVI. Map distances between two consecutive markers varied from 0 to 7.86 cM, while the gap average between markers varied from 1.37 cM in the LGI and 1.78 cM in the LGVII (Table 4) (Supplemental File 1).

In addition, 158 and 180 sequences from the DArTseq-derived markers were respectively BLASted with *M. truncatula* and *C. arietinum* genomes that, together with the 93 previously mapped SNPs markers, allowed to define the correspondence between LGs from *P. sativum* ssp. *syriacum* × *P. sativum* ssp. *sativum* cross and their *P. sativum* chromosome assignment, as follows: 26 SNP and 61 DArTseq-derived markers linked the LGI to the *P. sativum* LG3; 11 SNP and 66 DArTseq-derived markers linked LGII to the *P. sativum* LG7; 11 previously reported SNP markers, as well as 45 DArTseq-derived markers linked LGIII to LG4; 15 SNP and 51 DArTseq-derived markers related LGIV to LG2; 20 SNP and 34 DArTseq-derived markers linked LGV to the *P. sativum* LG6; 5 SNP and 35 DArTseq-derived markers linked LGVI to the *P. sativum* LG5; finally, 6 SNP and 46 DArTseq-derived markers linked the LGVII to the LG1 (Table 4) (Supplemental File 2).

### QTL’s analysis

**Figure 5:**
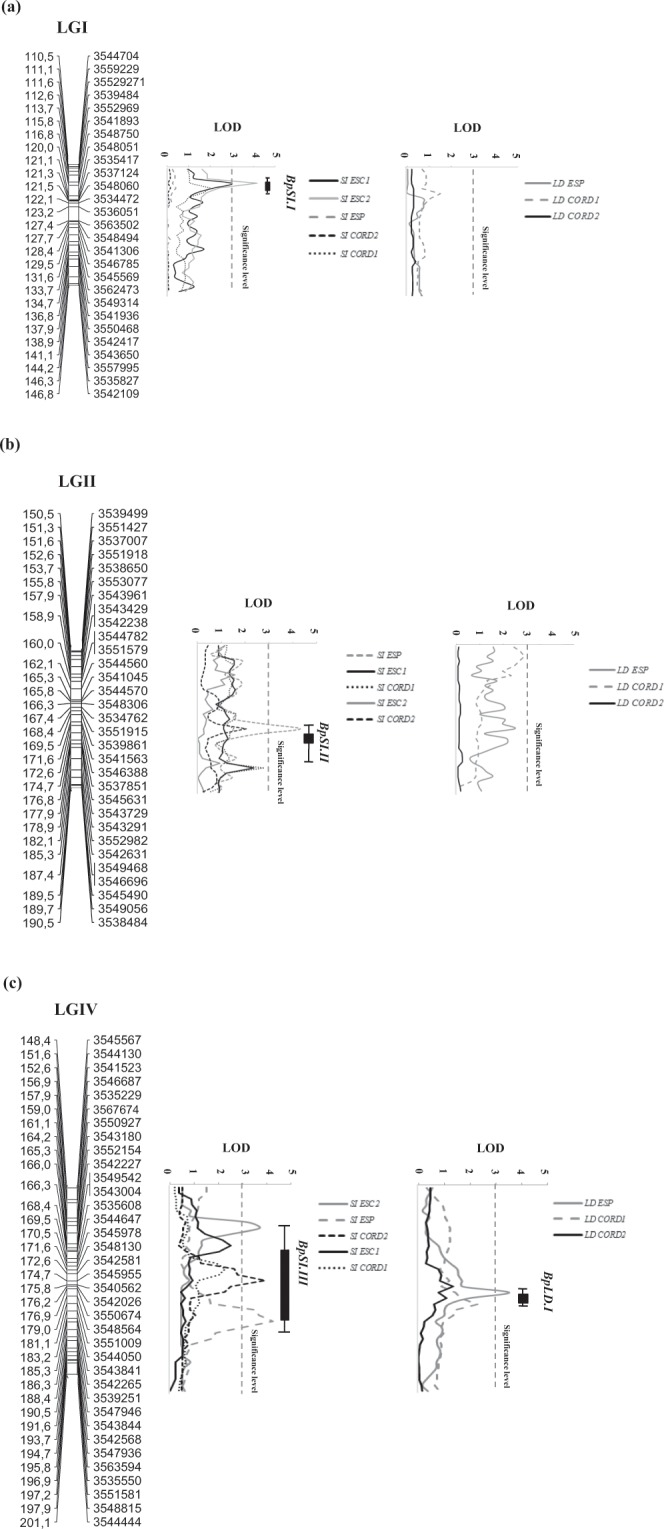
Likelihood plots of consistent quantitative trait loci (QTL) for adult plant resistance to weevil measured under field conditions in different year and environment on linkage groups (LG) I (**a**), II (**b**), and IV (**c**), using MapQTL in the P665 × Messire RIL population. Significant LOD thresholds were detected based on 1000 permutations. Absolute positions (in cM) of the molecular markers along LGs are shown on the vertical axes. SI ESC1, SI ESC2, SI ESP, SI CORD1, SI CORD2: seed infection (%) under field conditions measured at Escacena del Campo (Spain) during seasons 2013/2014 and 2014/2015, at Espiel (Spain) during season 2015/2016, and at Córdoba (Spain) during growing seasons 2014/2015 and 2015/2016; LD ESP, LD CORD1, LD CORD2: larval development measured at Espiel (Spain) during season 2015/2016, and at Córdoba (Spain) during growing seasons 2014/2015 and 2015/2016. QTL locations are represented as 1-LOD bars and 2-LOD whiskers on the linkage maps.

Both CIM and MIM analyses yielded similar results, therefore only CIM results are reported here. Quantitative trait loci analysis with CIM revealed genomic regions located in LGs I, II and IV involved in weevil resistance assessed as reduction of SI and LD. Even when QTLs where not significant in all environments studied, they showed a similar tendency albeit with low LOD values (Fig. 5).

Three QTLs associated to reduced SI (named *BpSI.I*, *BpSI.II* and *BpSI.III*) explained individually from 14.8 to 24.3% of weevil SI variation depending on the environment and together from 15 to 44.5%. *BpSI.I* was significant at ESC1 and ESC2 environments, with LOD score of 3.00 and 4.2 and explaining 14.8 and 24.3% of SI variation, respectively. *BpSI.I* peak was localized for both environments scored at 112.6 cM from the beginning of the LGI, between the DArT markers 35529271 and 3552969 (Table 5; Fig. 5). The distance to the left and to the right flanking markers was of 1.05 cM, respectively. *BpSI.II*, showed a LOD score of 4.3 and explained 19.2% of the phenotypic variation under ESP environmental conditions (Table 5; Fig. 5). *BpSI.II* was localized at 169.5 cM from the beginning of LGII, between DArT markers 3551915 and 3541563. The distance to the left and to the right flanking markers was of 1.05–2.1 cM, respectively. Interestingly, similar peaks were found in the same region (at 185.2 cM) for ESC1 and CORD1 environments but with LOD values under the significant threshold (LOD 2.3 and 2.6 respectively). *BpSI.III* showed LOD scores of 3.7, 4.3 and 3.9 for ESC2, ESP and CORD2 environments, respectively. *BpSI.III* explained from 18.4 to 22% of the phenotypic variation (Table 5; Fig. 5). *BpSI.III* was localized between 164.2 and 183.2 cM from the beginning of LGIV, between the derived DArT markers 3537674 and 3542227 (for SI ESC2), 3551009 and 3543841 (for SI ESP) and markers 3548130 and 3545955 (for SI CORD2) (Table 5). The distance to the left and to the right flanking markers was lower than 2 cM for each side. Similarly to the previous QTL, peaks in this same region were also detected for SI ESC1 and SI CORD1 (located at 162.1 and 175.8 cM, respectively), explaining 12.7 and 11.5% of the variation, but with no significant LOD scores (2.3 and 2.0 values, respectively).

**Table 5:** Position and effects of quantitative trait loci (QTL) for plant resistance to *B. pisorum* based on percentage of seed infection (SI) and larval development (LD) measured in field over five different environments using composite interval mapping (CIM) by Windows QTL Cartographer v2.5 in the P665 × Messire RIL population.

QTL^a^	LG^b^	Trait^c^	Peak^d^	Flanking markers		LOD^e^	Add^f^	R^2g^
*BpSI.I*	I	SI ESC1	112.6	35529271	3552969	3.0	−4.42	14.8
		SI ESC2	112.6	35529271	3552969	4.2	−0.24	24.3
*BpSI.II*	II	SI ESP	169.5	3551915	3541563	4.3	−0.48	19.2
		SI ESC1*	185.26	3552982	3549468	2.3	−0.13	7.3
		SI CORD1*	185.26	3552982	3549468	2.6	−0.27	9.1
*BpSI.III*	IV	SI ESC2	164.22	3537674	3542227	3.7	−0.22	22.0
		SI ESP	183.20	3551009	3543841	4.3	−0.46	19.0
		SI CORD2	172.64	3548130	3545955	3.9	−0.42	18.4
		SI ESC1*	*162.12*	3542500	3542038	*2.3*	−*0.16*	*12.7*
		SI CORD1*	*175.8*	3545955	3542026	*2.0*	−*0.30*	*11.5*
*BpLD.I*	IV	LD ESP	175.8	3545955	3542026	3.5	0.12	16.1
		LD CORD1*	*176.8*	3543378	3550725	2*.3*	*0.03*	*7.5*

^a^QTL that extend across single one-log support confidence intervals were assigned the same symbol.

^b^LG linkage group.

^c^SI CORD1, SI CORD2, SI ESC1, SIESC2 and SI ESP: seed infection (%) under field conditions measured at Córdoba (Spain) during growing seasons 2014/15, 2015/16, at Escacena del Campo (Spain) during seasons 2013/14 and 2014/15 and at Espiel (Spain) during season 2015/16, respectively.

^d^*Peak* QTL position (cM).

^e^*LOD* the peak LOD score.

^f^*Add* the additive effect.

^g^*R*^*2*^ proportion of phenotypic variance explained by the respective QTL (%).

^*^QTL following the same tendency but with no significant LOD values.

A single QTL associated with reduced LD was identified in LGIV with a LOD score of 3.5. *BpLD.I* explained 16.1% of the phenotypic variation for LD at ESP and was located at 175.8 cM from the beginning of the LGIV, between flanking markers 3545955 (1.05 cM) and 3542026 (1.76 cM) (Table 5; Fig. 5). In the same region a peak was found in LD CORD1 (located at 176.8 cM), explaining 7.5% of the variation, but with no significant LOD score (2.3). This was not observed in the third environment (CORD2).

The resistance-enhancing allele for SI came from the resistant parent P665, as indicated by the negative additive genetic effects (values from −4.42 to −0.22) (Table 5). By contrary, reduced LD derived from susceptible parent Messire (additive effect = 0.12) (Table 5).

The sequences of the markers linked to the QTLs identified were checked in the pea transcriptome assembly available online^32^ revealing seven transcripts (Supplemental File 3) linked to potential candidate genes within the genomic regions involved in weevil resistance in pea, as follows: two candidate genes were identified in LGI genomic region (corresponding to DArT markers 3546831 and 3551908); three candidate genes were identified in LGII genomic region (corresponding to DArT markers 3548194, 3552459 and 3549249); finally, two candidate genes were identified in LGIV genomic region (corresponding to DArT markers 3549680 and 3548130) (Supplemental File 3).

## Discussion

Peas seed yield and quality losses caused by pea weevil worldwide reinforce the need to develop resistant cultivars. This is hampered by the scarcity of sources of resistance and the lack of knowledge on its inheritance. Some sources of incomplete resistance are available, but mainly in wild relatives or on non-elite germplasm, forcing for a crossing and thorough selection what is further complicated by environmental influences on insect life cycle and infestations. Availability of molecular markers is most needed to facilitate this selection. Moreover, the possibility to combine different QTLs conferring resistance to a given pest or disease, or to different diseases, in the same cultivar is an added value of the recognition of QTLs associated to pea resistance^34^.

This recently constructed integrated genetic linkage map developed with an advanced RIL population (F_8:9_) from an interspecific cross (*P. sativum* ssp. *syriacum* × *P. sativum* ssp. *sativum*) and genotyped by DArTseq technology consists of seven linkage groups (LGs) covering a total length of 2,503.6 cM, with an average concentration of 2.61 markers cM^−1^ and an average marker-marker gap distance of 1.49 cM. Totally, 6,447 DArTseq-derived markers were polymorphic on the panel of 108 RIL populations, showing a good polymorphic information content (PIC) average value of 0.44 (values rating from 0.34 to 0.51), which indicates that those markers should be considered of importance^20^. In addition, 93 previously published SNP markers were mapped (working as reference markers) and were used as a bridge allowing us to determine the orientation of the linkage groups found and connect our linkage map with recently published *P. sativum* consensus maps ^29,35–37^. We found a general consistency on the markers relative position and order found in the present work and those reported in previously published *P. sativum* maps. In addition, we also found consistency between the total genetic lengths of the present map (2,503.6 cM) with respect to other recognized *P. sativum* maps, as example those by Timmerman-Vaughan *et al*.^38^ (with 2,416.7 cM) and more recently by Sudheesh *et al*.^39^ (2,555 cM). Over 74% of the 6,540 mapped markers showed marker-marker linkage tendency (in groups of at least two markers), which ended into 1699 bins. These markers represent probably mostly gene-rich regions, as DArT method of complexity reduction targets the hypomethylated regions of the genome. This is in agreement with previous observations found in the genetic maps of other several species, such as chickpea, rapeseed^21,40^ and, more recently, *P. fulvum*^22^ as example. The distribution of marker was uniform along the whole map, as demonstrated by the marker density (distance between 2 markers) which ranged from 1.37 and 1.78 cM (on LGI and LGVII, respectively). These rates are far higher than those reported on recent *P. sativum* published genetic maps ^29,39^, suggesting that the entire genome is particularly suitable to select markers useful for their application in marker assisted selection (MAS) and QTL detection, as well as in whole-genome breeding strategies, comparative mapping and/or genome organization studies^22^.

According to our knowledge, this is the first high-density integrated DArTseq SNP-based genetic map analysis for *Bp* resistance, even more using and advanced RIL population involving two subspecies, *sativum* and *syriacum*, under field conditions. Data from field phenotyping showed lower seed infection (SI) and larval development (LD) values in all environments for parental line P665 compared to Messire. These resistance traits showed a continuous distribution in the RIL population, indicating their quantitative nature of the inheritance. This is in accordance with previous studies in which partial resistance against *B. pisorum* was described in an interspecific cross between *P. sativum* ssp. *sativum* × *P. fulvum*^41^. For SI values, most of the RIL families revealing a level of resistance skewed toward low seed infection rates which suggest that the combination of both parental lines can enhance the level of resistance provided by the resistant accession P665. By contrary, LD values showed a level of resistance skewed toward high larval development rates, which is in agreement with findings from Aryamanesh *et al*.^41^.

Environmental conditions can affect both the insect and the plant life cycles^14,42^, resulting in a high influence of the environment on the expression of the QTL. This newly developed map allowed us to identify three QTL related to reduced SI and one related to LD, which were localized on LGs I, II and IV from our interspecific *Pisum* map, corresponding to the LGs 3, 7 and 2 of *P. sativum* genetic map, respectively^29,35,36^. *BpSI.I* (LGI) was significant in Escacena del Campo trials over the two seasons (ESC1 and ESC2). *BpSI.II* (LGII) was significant in Espiel environment (ESP). *BpSI.III* (LGIV) was significant in CORD2, ESC2 and ESP, while peaks although with non-significant LOD scores were also found for CORD1 and ESC1, suggesting the high implication of this pea genomic region in resistance to *Bp* infestation.

SI reduction could be influenced by several factors encompassing multiple plant resistance mechanisms that can act separately or simultaneously. Accession P665 showed pigmented flowers and pods, what is associated to condensed tannins content that might deter *Bp* oviposition^43,44^. Also a possible presence of a thick wax layer or volatiles should be considered as responsible of seed infection^45,46^. Both factors will deserve further studies in a near future.

In the same region of LGIV, a QTL (named *BpLD.I*) was also detected for LD resistance in ESP environment. Interestingly a peak with non-significant LOD value was also found in CORD1 environment. This suggests the possibility that more than one gene were located in the same region of the LGIV explaining both characters (SI and LD). We found that the QTL associated to reduced SI and LD identified in LGIV were closely located to the *HRIP_SNP1* marker, localized at 152.64 cM, which is implicated in the response to HR lesion-inducing like protein (*HRIP*)^47^. A further search for potential candidate genes based on the molecular markers in the region of this QTL highlighted the transcripts PsCam020860 (IPR009003: Serine/cysteine peptidase, trypsin-like; 145.3 cM) and PsCam010880 (IPR023210: Aldo/keto reductase; 171,6 cM) of the Unigene set of pea^32^. PsCam020860 corresponds to the DArT marker 3549680 due to its homology to a serine-type peptidase expressed in common bean and related with the disease resistance (R) gene cluster B4, highly expressed in pea leaves and shoots^48^. PsCam010880 corresponds to the DArT marker 3548130 due to its homology to an aldo/keto reductase expressed in pea in shoots, upper leaves and apical nodes which have been described to play a capital role in detoxifying the cytotoxic reactive aldehydes during stress responses from biotic and abiotic origin^49^. This agreed with previous studies, that reported in the same region the presence of multiple QTLs controlling bleaching resistance in pea^50^, as well as pea seed-borne yellow mosaic virus and pea aphid resistances^51^. Pea colored seeds have been associated with a higher free phenolic acid content and condensed tannins, which could affect *Bp* LD, as happen for other pests as *Callosobruchus chinensis* and *C. maculatus*^52,53^. It has been previously described that accession P665 also displayed antibiosis by hampering larval development^14^. This trait could be affected by a range of compounds that could be toxic for *Bp* larvae. The huge variability found along the environments could be possibly linked to the biochemical differences suffered by plants in different environments, as mentioned above.

QTL *BpSI.I* was located in LGI (corresponding with LG3 from *P. sativum*), in correspondence with DArT markers 3546831 and 3551908. Sequence from marker 3546831 corresponds to the PsCam056412 transcript (IPR010525: Auxin response factor, *ARF*; 115.8 cM) homologous to the protein *AtNAC2* of *A. thaliana*, while marker 3551908 corresponds to the PsCam036732 transcript (PsCam036732: Pentatricopeptide repeat, *PPR*P; 115.8 cM) homolog with the protein *At5g48910* of *A. thaliana*, both highly expressed in the apical nodes and upper plant leaves. Both proteins are involved in diverse and crucial roles affecting plant growth and development which include embryogenesis, chloroplast development and signaling in case of stress responses^54^. *PPRP* is expressed in light and sugar-induced stress responses, in callose-mediated defense, ROS-signaling, and resistance against fungal infection^55^. Auxins are strongly involved in biotic stress responses through *ARF*s^56^. Interestingly, auxins were reported to be upregulated in our *Bp* resistant parental line P665 after infection with the fungal pathogen *Didymella pinodes* and *ArfB3* has been proposed as candidate gene for resistance to *D. pinodes* in pea^16,29^.

Finally, QTL *BpSI.II* was correlated with LG7 of *P. sativum*, where markers 3548194, 3552459 and 3549249 were identified as potential candidate genes. DArT marker 3548194 corresponds to the PsCam045147 transcript (IPR000767: Disease resistance protein; 165.3 cM). DArT marker 3552459 corresponds to the PsCam056343 transcript (IPR000767: Disease resistance protein; 172.6 cM), respectively similar to the proteins *At5g6302*0 and *At3g14460* of *A. thaliana*. Both proteins are considered as disease resistance proteins belonging to the nucleotide-binding site-leucine-rich repeat (NBS-LRR) family involved in pathogen sensing mechanisms, as well as in defense responses and apoptosis^57,58^. DArT marker 3549249 corresponds to the PsCam052343_1_AA transcript (IPR001424: Superoxide dismutase SOD, copper/zinc binding domain; 174.7 cM), which is highly expressed in pea leaves, tendrils and stems. SODs are one of the most efficient enzymatic antioxidant defences of eukaryotes that strongly reduce the formation of toxic and highly reactive oxidant molecules to protect cells against oxidative injury. Increases in SOD activity have been observed in plants in response to treatment with herbicides, drought, chilling, anoxia, pathogens and pests injuries. In fact, high SOD activity has been recently found in resistant rice variety against the brown plant hopper (*Laodelphax striatellus*)^59^. In addition the QTL *BpSI.II* was also closely located with the gene *ppi2* involved in the resistance in pea to *P. syringae* pv. *pisi* race 2 as well as with the markers *Selbin_SNP2* (171.6 cM) and *Dolf4_SNP3* (167.4 cM)^60^. The first one is associated to a protein that binds to selenium and is also involved in senescence and stress response, between others, while *Dolf4* is a plant-specific DOF (DNA-binding with One Finger)-type transcription factor, which regulate several biological processes such as responses to light and phytohormones, seed maturation and germination, between others^61^. All these factors could directly affect the *Bp* larvae on their way to the seed influencing seed infestation levels.

In conclusion, this study reveals one robust QTL associated to reduced SI values with significant LOD scores in most of the environments studied. In addition, two additional QTL associated with low SI have also been identified as well as several genetic markers associated to low pea infestation which are of huge interest to preserve the pea seed intact. Finally, a QTL associated to reduce LD was also found. Importantly, the identification of potential candidate genes is the initial step needed before clone the genes involved in *B. pisorum* resistance in pea. Additional studies such as functional analyses are planned in a near future to validate their role in resistance.

## Supplementary information

Dataset 1.

Dataset 2.

Dataset 3.
